# The ‘Health Collapse Spiral’: A 35‐Month Longitudinal Case Study of Physiological Reserve Depletion in a Centenarian Following COVID‐19

**DOI:** 10.1002/ccr3.72965

**Published:** 2026-06-18

**Authors:** Shan Xu, Wei Zhang, Qiaoxiang Yin, Jie Wei, Yanmin Ma, Wenwen Jia

**Affiliations:** ^1^ Department of Geriatrics Air Force Medical Center Beijing China

**Keywords:** centenarian, COVID‐19, frailty, health collapse spiral, longitudinal case study, physiological reserve

## Abstract

This 35‐month case report documents irreversible physiological reserve depletion in a centenarian multimorbid man after COVID‐19. Prior to infection, he maintained precarious homeostasis for 17 years despite severe frailty (CFS 7), with stable CKD stage 3a (eGFR 58.3 mL/min/1.73 m^2^) and albumin 36.2 g/L. Acute COVID‐19 triggered a health collapse spiral (a stepwise, irreversible multisystem deterioration driven by cumulative stressors), characterized by: eGFR decline > 60% (58.3 to 22.4 mL/min/1.73 m^2^), albumin drop to 28 g/L, CRP surge > 360‐fold (peak 254.2 mg/L), Barthel Index loss from 80 to 0, and predominant recurrent MDR/PDR *Pseudomonas aeruginosa* infections (42.9%). Death followed palliative transition. This case suggests that acute infections irreversibly disrupt centenarian homeostasis, with eGFR, albumin, and CRP serving as reliable reserve proxies, and that reserve‐centered management is essential for this population.

**Key Clinical Message**

COVID‐19 triggers an irreversible “health collapse spiral” in centenarians; eGFR, albumin, and CRP are dynamic proxies for physiological reserve, requiring reserve‐centered management via longitudinal monitoring, advance care planning, and avoidance of futile interventions.

## 1 Introduction

Global demographic aging has made physiological reserve depletion and multisystem vulnerability central challenges in modern medicine [1] challenges exemplified by frailty and multimorbidity, which increase susceptibility to physiological stressors. This vulnerability was underscored by COVID‐19's disproportionate impact on older adults [2, 3]. While short‐term outcomes in this population are well‐documented, granular longitudinal data tracing the clinical trajectory from acute infection to death among the oldest old, particularly centenarians, remain critically scarce [Figure 1].

**FIGURE 1:**
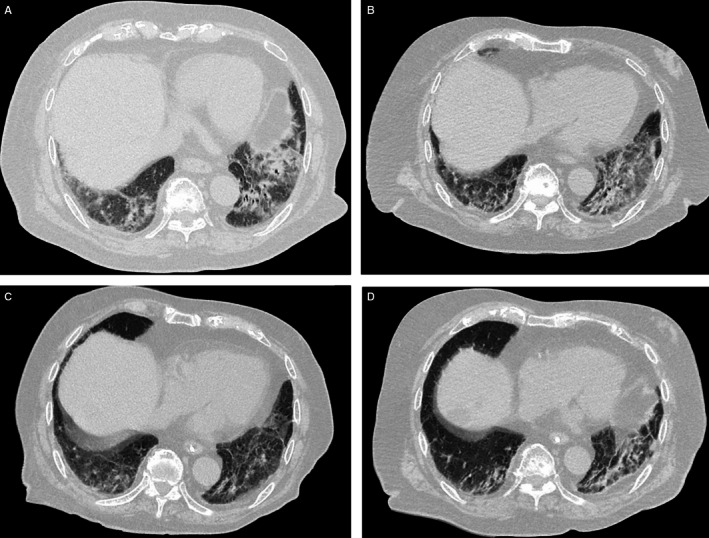
Longitudinal Evolution of Chest CT Findings Over the 35‐Month Clinical Trajectory: Serial chest CT images demonstrate progressive pulmonary injury aligned with the patient's health collapse spiral and core clinical phases (consistent with Table 1 and Discussion). Time intervals (relative to index COVID‐19) and key milestones: (1A) Trigger Event Phase (December 2022, 0 months): Acute phase of severe COVID‐19 pneumonia, characterized by bilateral diffuse ground‐glass opacities involving > 50% of lung parenchyma, disrupting 17 years of precarious homeostasis; (1B) Acceleration Phase (June 2023, 6 months): Organizing pneumonia with patchy consolidations and interstitial thickening; (1C) Vicious Cycle Phase (April 2024, 16 months): Revealed insidious pulmonary fibrosis, characterized by bilateral reticular opacities, interlobular septal thickening—radiological features reflecting cumulative lung injury from recurrent infections; (1D) Pre‐Systemic Collapse Phase (July 2025, 31 months): End‐stage lung disease with superimposed acute infection, showing new focal parenchymal consolidation superimposed on a background of established pulmonary fibrosis. These radiological changes directly parallel the synchronous decline of eGFR, albumin, and CRP in Table 1.

This report addresses this gap by presenting a 35‐month progression of a centenarian man from post‐COVID‐19 survival to multisystem failure. We conceptualize his decline as a health collapse spiral—a stepwise, irreversible multisystem deterioration driven by cumulative stressors that exhaust physiological reserve, culminating in functional collapse and death. Within the framework of stress‐induced frailty decompensation [4, 5], this case offers a rare longitudinal visualization of systemic physiological reserve depletion at the extreme limits of human age.

## 2 Case Report

This study was conducted under routine clinical management, with protocol approval from the Institutional Ethics Committee of the Air Force Medical Center (No. 2022–224‐YJ01) and written consent obtained from the patient's legally authorized representative.

In October 2022, a comprehensive geriatric assessment (CGA) was performed in a 102‐year‐old male patient, including medical history (unvaccinated), cognitive function (Mini‐Mental State Examination [MMSE], not longitudinally assessed), functional status (Clinical Frailty Scale [CFS] and Barthel Index [BI]), nutritional assessment, medication review, and social‐lifestyle evaluation [6]. Retrospective review of electronic health records (January 2005–October 2022) confirmed long‐term pre‐infection baseline stability.

The patient was a widower, lifelong non‐smoker and non‐drinker who resided with a caregiver. He was on long‐term medications—including nitrate vasodilators, statins, anti‐osteoporosis agents, and endocrine therapy for prostate cancer—without adverse effects. He carried 31 distinct diagnoses, including atherosclerotic coronary heart disease, chronic kidney disease (CKD) Stage 3a, type 2 diabetes, hormone‐refractory prostate cancer (post‐orchiectomy), laryngeal squamous cell carcinoma (radiotherapy alone), chronic bronchitis, severe frailty (CFS 7), and sarcopenia (SARC‐F [Sarcopenia Assessment Tool] score ≥ 4) [7].

Prior to SARS‐CoV‐2 infection, he maintained independence in basic activities of daily living (ADL) (BI 80) but required partial assistance with instrumental activities of daily living (IADL). Renal function, albumin, and CRP had remained stable for 17 years, reflecting “precarious homeostasis” [1, 4].

### 2.1 Trigger Event and Post‐Infection Decline (The ‘Trigger Event Phase’ in Table 1; December 2022)

**TABLE 1:** Longitudinal clinical trajectory (october 2022–November 2025).

Phase	Time	Key Clinical Event	Primary pathogen & Resistance profile	Main antimicrobial/antiviral regimen	Renal function (SCr [μmol/L] / eGFR [mL/min/1.73m2])	Albumin [g/L]	CRP [mg/L]	Functional status (BI/CFS)	Respiratory & supportive notes
Baseline	2022–10	Stable CKD stage 3a	—	—	90.2/58.3	36.2	0.7	80/7	None
Trigger Event	2022–12	First COVID‐19 pneumonia	SARS‐CoV‐2 (severe, Ct 29)	Nirmatrelvir/ritonavir (150/100 mg bid, 5 days); methylprednisolone; meropenem	75 → 81/65.2 → 58.1	36.2 → **32.4**	0.7 → **27.2** → 1.4	45/7	HFNC (high‐flow nasal cannula, FiO_2_ 0.4–0.6)
Acceleration Phase	2023–05 to 06	Second COVID‐19 pneumonia	SARS‐CoV‐2 (Ct 25); *K. pneumoniae* (Carb‐S)	Nirmatrelvir/ritonavir; methylprednisolone; meropenem	101.9 → 70.7/52.7 → 66.7	40.4 → **28.7**	0.8 → **10.7** → 4.2	45/7	HFOT (high‐flow oxygen therapy, FiO_2_ 0.35–0.5)
Vicious Cycle Phase	2023–07	First LRTI (lower respiratory tract infection)	*E. meningoseptica* (Levo‐S)	Meropenem → levofloxacin	70.7 → **125**/66.7 → **43.0**	33.7 → **31.2**	4.2 → **141.3** → 4.7	20/8	Type II respiratory failure; nocturnal NIV (non‐invasive ventilation) started; EN (enteral nutrition) initiated (1 kcal/mL)
	2023–08	Second LRTI	*S. maltophilia* ; *S. aureus*	Meropenem	81 → **115.2** → 106/61.7 → **48** → 51.1	40.2 → **38.3**	**65.9** → 1.3	20/8	NIV (8 h/night) + HFOT (12 h/day, FiO_2_ 0.4–0.55)
	2023–09	Third LRTI	Mixed flora (no dominant pathogen)	Meropenem	**136.4** → 93/**42.2** → 55.8	43.6 → **35.8**	1.9 → 2.6	25/8	HFOT (FiO_2_ 0.35–0.45); EN intensified (1 kcal/mL)
	2024–06 to 07	Fourth LRTI	Mixed flora (no dominant pathogen)	Cefoxitin → meropenem → ceftazidime+vancomycin+caspofungin → imipenem	**139.5** → 97/**40.6** → 53.4	35 → 39.2 → **33.1**	2.6 → **6.6**	20/8	Prolonged HFOT (FiO_2_ 0.45–0.6); EN intensified (1.8 kcal/mL)
	2024–08 to 09	Fifth LRTI	*p. aeruginosa* (MDR; CEPH‐R/FQ‐R)	Doxycycline (per AST)	**177.1** → **214.5**/**33.5** → **29.0**	35 → 41.3 → 35	6.3 → 5.7	15/8	Continued support (HFOT + intermittent NIV); EN maintained
	2024–10 to 2025–01	Sixth LRTI	*p. aeruginosa* (MDR; CARB‐R/FQ‐R)	Meropenem → ceftazidime → meropenem (rotated)	**181.4** → **224.7**/**33.0** → **27.9**	36.3 → **32.0** → 35.1	6.3 → **18** → 1.2	10/8	Progressive decline; continuous NIV + HFOT; EN maintained
	2025–05 to 08	Seventh LRTI	*p. aeruginosa* (MDR; CEPH‐R/FQ‐R)	Meropenem (multiple courses) → ceftazidime (rotated)	**208.9** → 163/**29.9** → 36.3	35.1 → 44.2 → 41.9	0.6 → **6.5** → 3.1	< 5/9	Increased infection frequency; continuous NIV; EN maintained with intermittent albumin support
Systemic Collapse Phase	2025–09 to 10	PDR *p. aeruginosa* bacteremia (terminal)	*p. aeruginosa* (PDR)	Meropenem+levofloxacin → linezolid+fluconazole → imipenem	**219.2** → **287.5** → **309**/**28.6** → **23.2** → **22.4**	41.9 → **28.0**	2.4 → **131.0**	0/9	Palliative transition; low‐flow O_2_ + symptom control; moderate malnutrition; EN for comfort
	Late 2025	Death	NA	NA	—	—	**254.2**	0/9	—

*Note:* Data are presented in chronological order from baseline to final assessment over the 35‐month study period. Values in bold indicate clinically significant changes (exceeding pre‐COVID‐19 baseline or progressing to advanced CKD/malnutrition). Pathogens were identified via culture and antimicrobial susceptibility testing (AST). eGFR was calculated using the CKD‐EPI equation.

Abbreviations: AST, antimicrobial susceptibility testing; CARB‐R, carbapenem‐resistant; CEPH‐R, cephalosporin‐resistant; Ct, cycle threshold; EN, enteral nutrition; FiO_2_, fraction of inspired oxygen; FQ‐R, fluoroquinolone‐resistant; HFNC, high‐flow nasal cannula; HFOT, high‐flow oxygen therapy; LRTI, lower respiratory tract infection; MDR, multidrug‐resistant; NIV, non‐invasive ventilation; PDR, pandrug‐resistant; RF, respiratory failure; SCr, serum creatinine.

In December 2022, the patient presented with persistent fever, productive cough, and exertional hypoxemia (room‐air peripheral capillary oxygen saturation [SpO_2_] 85%). Severe COVID‐19 pneumonia was confirmed by positive SARS‐CoV‐2 reverse transcription‐polymerase chain reaction (RT‐PCR) (cycle threshold [Ct] 29) and chest computed tomography (CT) showing bilateral ground‐glass opacities involving > 50% of lung parenchyma.

Initial management included nirmatrelvir/ritonavir (150/100 mg twice daily [bid] for 5 days) adjusted for Stage 3a chronic kidney disease (CKD), intravenous methylprednisolone (40 mg/day, tapered over 5 days), meropenem, and high‐flow nasal cannula oxygen (fraction of inspired oxygen [FiO_2_] 0.4–0.6) to maintain SpO_2_ ≥ 95%. Viral clearance was achieved on hospital day 7 (RT‐PCR Ct > 35).

Despite virological cure, profound functional and biochemical declines emerged: BI dropped from 80 to 45, accompanied by elevated inflammation and early malnutrition. Although serum creatinine initially decreased transiently (attributed to acute volume depletion), it rebounded by February 2023, ending 17 years of renal stability. This acute COVID‐19 terminated the patient's prolonged precarious homeostasis and thus triggered multisystem decline [1, 4].

### 2.2 Acceleration Phase (The ‘Acceleration Phase’ in Table 1; May–June 2023)

In May 2023, the patient developed dyspnea and fever, with COVID‐19 reinfection confirmed by RT‐PCR (Ct: 25). Treatment mirrored the initial episode, with virological clearance on hospital day 9. June 2023 chest CT showed organizing pneumonia with patchy consolidations and interstitial thickening.

Renal function remained stable, but inflammation and malnutrition worsened—leading to the initiation of oral nutritional supplements and weekly inflammatory marker monitoring. Notably, swallowing dysfunction emerged by June 2023 (occasional coughing, ~30% intake reduction), raising concerns about aspiration risk.

### 2.3 Progressive Decline and Functional Collapse (The ‘Vicious Cycle Phase’ in Table 1; July 2023–August 2025)

In July 2023, an aspiration event precipitated acute‐on‐chronic type II respiratory failure (pH 7.21; PaCO_2_ 73 mmHg; PaO_2_ 58 mmHg), necessitating nocturnal NIV and high‐flow nasal cannula oxygen. The patient subsequently became completely functionally dependent (BI ≤ 30), requiring 24‐h care and nasogastric tube feeding. This acute insult triggered synchronous multisystem decompensation consistent with a “scissor effect”: Inflammatory markers surged while albumin fell, accompanied by deterioration to CKD Stage 3b—marking the failure of physiological compensatory mechanisms [1, 8].

Over the subsequent 26 months, seven LRTIs occurred (42.9% MDR *Pseudomonas aeruginosa* ), consistent with post‐COVID‐19 antimicrobial resistance [9, 10]. Antimicrobial selection was guided by serial culture and susceptibility testing, with de‐escalation to narrower‐spectrum agents whenever feasible, in keeping with antimicrobial stewardship principles (Table 1).

A 7‐month stability period (Oct 2023–May 2024) was achieved with high‐flow oxygen therapy and nutritional support (albumin > 35 g/L, no infections). However, surveillance CT in April 2024 revealed insidious pulmonary fibrosis, characterized by bilateral reticular opacities, interlobular septal thickening—radiological features reflecting cumulative lung injury from recurrent infections [11].

Despite targeted supportive care (enteral nutrition, intermittent albumin supplementation), renal function and albumin demonstrated progressive stepwise decline without returning to baseline. By August 2025, renal function had persisted in CKD Stage 3b; albumin remained below 35 g/L, and the CFS score had deteriorated to 9 (terminally ill). July 2025 chest CT demonstrated end‐stage lung disease with acute superimposed infection upon background fibrosis—consistent with the seventh MDR *P. aeruginosa* infection. This trajectory illustrates the terminal health collapse spiral—characterized by irreversible physiological reserve depletion and complete loss of resilience to stressors—as conceptualized in frailty research [1, 4].

### 2.4 End‐Stage and Transition to Palliative Care (The ‘Systemic Collapse Phase’ in Table 1; September–November 2025)

In September 2025, the patient developed septic shock from pandrug‐resistant (PDR) *P. aeruginosa* bacteremia (blood culture‐confirmed). Catastrophic multisystem failure ensued: CRP peaked at 254.2 mg/L, albumin fell to 28 g/L (moderate malnutrition), renal function deteriorated to eGFR 22.4 mL/min/1.73 m^2^ (SCr 309 μmol/L), hypercapnic respiratory failure decompensated (pH 7.18, PaCO_2_ 95 mmHg, PaO_2_ 52 mmHg), and hemodynamic instability required vasopressors.

Management aligned with the patient's December 2022 advance directive (declining invasive life‐sustaining measures). Following multidisciplinary consultation, care transitioned to palliative care focused on symptom control and dignity preservation.

The patient died peacefully in late 2025 after a palliative transition. His trajectory reflects end‐stage physiological reserve depletion—initiated by index COVID‐19, perpetuated by recurrent infections, and overwhelmed by terminal PDR bacterial insult—exemplifying the health collapse spiral in extreme aging, where cumulative stressors exceed compensatory capacity [1, 4].

## 3 Discussion

The 35‐month trajectory detailed above illustrates a progressive, irreversible decline following acute COVID‐19—a pattern characteristic of the health collapse spiral in a multimorbid centenarian, rooted in the classic geriatric theory of physiological reserve depletion [1]. Notably, this centenarian, with 31 comorbidities and severe frailty (CFS 7, BI 80, pre‐infection albumin 36.2 g/L; Table 2), was inherently vulnerable to stressor‐induced decompensation. COVID‐19 acted as a proximate accelerant of physiological reserve depletion rather than its sole determinant. Specifically, the patient's stepwise multisystem decline demonstrates systematic reserve exhaustion within the frailty decompensation paradigm [4, 5], suggesting that physiological reserve—not baseline disease burden—may serve as a key prognostic indicator for outcomes at the human lifespan's limits [1, 2].

**TABLE 2:** Baseline clinical characteristics (prior to december 2022).

Domain	Specific disease/feature/parameter
Cardiovascular	Atherosclerotic coronary heart disease; Chronic heart failure (NYHA class II); Severe postprandial hypotension; Hyperlipidemia; Paroxysmal tachycardia
Renal & Metabolic	Chronic kidney disease (stage 3a); Type 2 diabetes mellitus (diet‐controlled)
Oncological History	Hormone‐refractory prostate cancer (post‐orchiectomy); Laryngeal squamous cell carcinoma (radiotherapy alone, non‐resected)
Respiratory	Chronic bronchitis
Musculoskeletal	Severe degenerative osteoarthritis; Sarcopenia (SARC‐F score ≥ 4)
Geriatric Syndromes	Severe frailty (CFS score: 7); Mild cognitive impairment (MMSE score: 24/30); No significant depressive symptoms (GDS score: 4/15)
Functional Status	Partial dependence for IADL; BI score: 80; COVID‐19 vaccination (unvaccinated)
Long‐term Stable Laboratory Parameters	Serum creatinine: 90.2 μmol/L; eGFR: 58.3 mL/min/1.73 m^2^; Albumin: 36.2 g/L; CRP: 0.7 mg/L

*Note:* The total of 31 diagnoses includes minor age‐related conditions (e.g., dental caries, post‐cataract surgery, hearing loss). Laboratory values were obtained during the comprehensive geriatric assessment in October 2022, representing the patient's pre‐infection baseline. These parameters had remained stable within clinically comparable ranges for approximately 17 years prior to this assessment.

Abbreviations: BI, Barthel Index; CFS, Clinical Frailty Scale; CRP, C‐reactive protein; GDS, Geriatric Depression Scale; IADL, Instrumental Activities of Daily Living; MMSE, Mini‐Mental State Examination; NYHA, New York Heart Association; SARC‐F, Sarcopenia Assessment Tool.

Before SARS‐CoV‐2 infection, the patient's stable parameters reflected ‘precarious homeostasis’—a fragile equilibrium characteristic of advanced aging with multimorbidity [1]. However, decades of such stability left him vulnerable to irreversible decompensation after acute infectious insults. We conceptualize extreme aging as a dynamic balance between intrinsic resilience and cumulative extrinsic stressors [2].

Severe COVID‐19 pneumonia precipitated progressive functional decline consistent with stressor‐induced decompensation in frailty [4]. We selected eGFR, albumin, and CRP as feasible, validated proxies for renal, nutritional, and inflammatory reserve, respectively [1, 7, 12]. Repeated physical assessments were impractical in this increasingly immobile patient, and specialized frailty markers (e.g., IL‐6, vitamin D) were not routinely available.

The synchronous deterioration demonstrates the ‘scissor effect’: Inflammatory activation (CRP surge) and nutritional exhaustion (albumin decline) progressed in opposite but mutually reinforcing directions [1, 8]. Persistent inflammation drives catabolism and impairs nutrient absorption, while malnutrition compromises immune function and tissue repair, thereby amplifying inflammatory dysregulation [11, 13]. This bidirectional feedback loop accelerates reserve depletion and reduces the efficacy of supportive care, highlighting the unique nature of the health collapse spiral in centenarians.

This pattern reflects the ‘network physiology’ framework [1] and stress‐induced decompensation model [4, 5], wherein virologically resolved infections may perpetuate subclinical organ injury [8, 11, 13]. Our trajectory integrates three geriatric frameworks: Frailty progression (CFS 7 → 9) [4, 5], post‐acute COVID‐19 syndrome [11, 13], and physiological resilience depletion (“scissor effect”) [1, 8], underscoring that centenarian outcomes emerge from dynamic stressor‐reserve interplay.

In centenarians, longitudinal organ function trajectories provide more prognostic insight than cross‐sectional measurements, given that extreme aging is characterized by impaired adaptive capacity [7, 12]. This case invites preliminary conceptualization of a ‘physiological reserve threshold’—the tipping point where cumulative stressors exceed remaining compensatory capacity, precipitating irreversible decompensation [1, 4, 8]. While transient declines below 35 g/L had occurred earlier, the patient's albumin fell to 31.2 g/L in July 2023, coinciding with initial functional collapse (BI ≤ 30) [7]; a subsequent eGFR decline to 22.4 mL/min/1.73 m^2^ (61.6% from baseline) accompanied end‐stage decompensation (BI = 0). Although definitive thresholds require validation in larger cohorts, these observations generate the hypothesis that early albumin < 35 g/L predicts impending functional decline [7], whereas an eGFR reduction > 50% from baseline or terminal albumin < 30 g/L (28.0 g/L in the present case) indicates irreversible physiological exhaustion in centenarians [12]. The clinical course unfolded a self‐perpetuating cycle of recurrent MDR/PDR infections, progressive renal dysfunction, malnutrition, and respiratory failure—mutually reinforcing drivers of irreversible reserve depletion and functional decline [1, 4, 9]. Critically, once physiological reserve is depleted beyond a threshold, aggressive organ‐supportive interventions become futile and may conflict with patient‐centered care, as illustrated by this patient's advance directive and transition to palliative care [6].

These hypothesis‐generating findings support a paradigm shift from reactive, organ‐siloed care to proactive, reserve‐centered management for multimorbid centenarians. Such strategies could incorporate baseline CGA [6], longitudinal surveillance of key physiological indices (eGFR, albumin, CRP, functional scales), iterative advance care planning [6], and early palliative care, with these approaches meriting further evaluation in prospective centenarian cohorts. This model minimizes crisis‐driven decisions, preserves patient autonomy, and addresses the specific vulnerabilities inherent to the health collapse spiral in extreme longevity. Unlike prior centenarian COVID‐19 studies limited to short‐term outcomes [2, 3], this 35‐month longitudinal observation provides rare empirical grounding for classic geriatric decline models—physiological reserve depletion [1] and frailty decompensation [4, 5], in the oldest‐old population.

As a single‐case observation, these findings are hypothesis‐generating and not generalizable to all centenarians. Additional limitations include the absence of longitudinal cognitive assessment despite baseline mild impairment (MMSE 24/30). Nevertheless, longitudinal biomarker trajectories illuminate reserve depletion as a mechanistic driver of mortality in extreme aging. Survival from acute infection may signal imminent health collapse among centenarians; reserve‐centered management thus emerges as an optimal approach to preserving dignity and autonomy.

## Author Contributions

**Shan Xu:** writing – original draft. **Wei Zhang:** conceptualization, funding acquisition, writing – review and editing. **Qiaoxiang Yin:** methodology. **Jie Wei:** data curation, formal analysis. **Yanmin Ma:** data curation, investigation. **Wenwen Jia:** project administration.

## Funding

This work was supported by the Clinical Research Program of Air Force Medical University (2022LC2202).

## Ethics Statement

The study protocol was approved by the Ethics Committee of the Air Force Medical Center (Approval No. 2022–224‐YJ01). The study was conducted in strict accordance with the ethical principles outlined in the Declaration of Helsinki and its subsequent amendments.

## Consent

Written informed consent for publication of clinical details, laboratory data, and chest CT images was obtained from the patient's legally authorized representative (daughter). The representative confirmed agreement to the open access publication of this case report.

## Conflicts of Interest

The authors declare no conflicts of interest.

## Data Availability

All data generated or analyzed during this study are included in this published article. Supporting Information (original laboratory test records and complete chest CT image sequences) are available to authorized users upon reasonable request.
